# Public Perception of Robot-Assisted Spine Surgery

**DOI:** 10.3390/jcm14134719

**Published:** 2025-07-03

**Authors:** Luca Fumagalli, Alexandros Moniakis, Alberto Pagnamenta, Andrea Cardia, Ivan Cabrilo

**Affiliations:** 1 Neurosurgery Department, Neurocenter of Southern Switzerland, Ente Ospedaliero Cantonale, 6900 Lugano, Switzerland; alexandros.moniakis@eoc.ch (A.M.); andrea.cardia@eoc.ch (A.C.); ivan.cabrilo@eoc.ch (I.C.); 2 Department of Intensive Care Medicine, Ospedale Regionale di Lugano, Ente Ospedaliero Cantonale, 6900 Lugano, Switzerland; alberto.pagnamenta@eoc.ch; 3 Clinical Trial Unit, Ente Ospedaliero Cantonale, 6900 Lugano, Switzerland; 4 Division of Pneumology, University Hospital of Geneva, 1205 Geneva, Switzerland

**Keywords:** spine surgery, robotic surgery, spinal instrumentation, navigation, enabling technology

## Abstract

**Background/Objectives**: The potential advantages of robotic assistance in spinal procedures are a growing area of interest, and patient perception plays a key role in its broader acceptance. However, public perception of robotic surgery in spinal operations remains unexplored. This study aims to assess the general public’s perceptions, expectations, and concerns regarding robot-assisted spine surgery. **Methods**: In the fall of 2024, a questionnaire was distributed to attendees at a public open day at the Neurocenter of Southern Switzerland, where the Globus ExcelsiusGPS™ spine surgery robot was demonstrated live on a mannequin. The 15-item questionnaire assessed demographic data, prior knowledge of medical robots, mental representations of surgical robots, expectations, and emotions after witnessing the demonstration. Data were analyzed using descriptive statistics, chi-square, Wilcoxon, McNemar tests, and logistic regression analysis. **Results**: A total of 109 questionnaires were collected. Most participants were female (64.4%) and had no direct experience with spinal pathology (79.8%). While 87.2% were aware of robotic surgery in general, only 65.1% specifically knew about its use in spine surgery. After witnessing the live demonstration, 81.9% felt reassured by the robot′s presence in surgery, compared to 61.3% before the demonstration (*p* = 0.007). Preference for robot-assisted surgery increased from 50.5% to 64.5% (*p* < 0.001). Notably, individuals with back-related issues showed greater confidence in the robot’s capabilities (*p* = 0.032). **Conclusions**: The general public perceives robotic spine surgery positively, viewing it as faster, more precise, and capable of performing tasks not readily performed by humans. The study highlights the importance of live demonstrations in enhancing trust and acceptance of robotic systems. Its findings have economic implications, as patients may be more likely to choose hospitals offering robot-assisted spine surgery. However, it is essential to also acknowledge alternative methods, such as computer-assisted navigation, which has demonstrated efficacy in spine surgery.

## 1. Introduction

Advances in medical technology have reshaped modern healthcare, with robotics emerging as a novel innovation in various surgical disciplines, including spine surgery. Robot-assisted surgery aims to improve precision, reduce surgical invasiveness and recovery times, and thereby enhance surgical outcomes. Yet, the integration of robotic systems in the operating room has elicited mixed reactions from the general public, ranging from enthusiasm [1] to skepticism [2].

Media has been shown to influence people’s perceptions of robots [3]. Science fiction books, movies, and television series often depict autonomous robots in dystopian contexts, triggering anxieties about machines replacing human control and decision-making. It can be assumed that these cultural representations contribute to public reluctance, as people project these fictional scenarios onto real-world technologies.

The transition from conventional freehand technique to robot-assisted spine surgery has unfolded over an extended period of time, progressively incorporating technological breakthroughs as they emerged. While freehand technique solely relies on surgeons’ anatomical knowledge and experience to guide screw placement, the use of intraoperative radiography and subsequently fluoroscopy provided an initial form of image guidance for screw insertion from the 1970s to the present day [4]. The advent of computer-assisted 3-dimensional navigation marked a significant advancement by enabling real-time visualization without direct sight and enhancing the precision of screw placement [5]. Robotic systems became commercially available from the 2000s, offering pre-planned trajectories and automating key steps previously dependent on manual execution in an attempt to further reduce screw placement inaccuracy [6,7]. A recent web-based survey conducted in 2023 among AO Spine members found that less than half regularly used spinal navigation and that one-third had never used a spinal navigation system, while only 19% identified themselves as robotic users [8], highlighting that although there is an evolution in the development of these technologies and methods, they remain widely in contemporaneous use.

The adoption of robotic systems in spine surgery therefore remains relatively recent [9,10] when compared to other better known medical robots such as the da Vinci Surgical System (Intuitive Surgical, Sunnyvale, CA, USA), used in abdominal, urological, and gynecological surgery. Consequently, there is still only limited comprehensive data on the long-term outcomes of robotic spine surgery. While early clinical studies suggest positive results [7,11], the novelty of the technology and the lack of extensive application may contribute to uncertainty over its potential advantages. The perspective of spine surgeons has been surveyed in this regard and viewed as positively impacting surgical training and practice [12,13], but the expectations and concerns of the general public regarding robot-assisted spine surgery have not yet been explored. Although the adoption and use of novel technology is ultimately driven by its clinical utility, other factors may influence its integration, such as its costs, surgeons’ willingness to adapt to new workflows, and patients’ views, as their involvement in care and management decisions increases.

Given the rapid pace of technological advancement and its potential benefits in spine surgery [7], understanding public perceptions of robotic surgery systems is essential—and particularly how these perceptions are shaped by demographics and personal background and experience—to ensure the successful integration of these technologies into clinical practice [14]. The aim of this study, therefore, is to investigate public perception of robot-assisted spine surgery for the first time.

## 2. Materials and Methods

This article follows the “Strengthening the Reporting of Observational Studies in Epidemiology (STROBE) Statement” checklist [15].

ChatGPT (version 3.5) was solely used to assist in English language editing, after the manuscript had been completed. All content, including the interpretations of results and conclusions, was generated and reviewed by the Authors alone. No AI tool was used to generate scientific content, analyze data, or draw conclusions.

### 2.1. Questionnaire and Survey

In the fall of 2024, the Neurocenter of Southern Switzerland held an open day for the general public. During this event, the neurosurgery department conducted a demonstration of the Globus ExcelsiusGPS™ spine surgery robot. Participants had the opportunity to observe the robot in action, interact with spine surgeons and company representatives, and operate the robot firsthand on an anatomical training model. A model-based explanation was also provided to clarify how the procedure is typically performed without robotic assistance.

A questionnaire was distributed to explore the general public’s knowledge, expectations, and concerns regarding the use of robots in spine surgery.

This 15-item questionnaire was divided into six sections: (1) demographic data; (2) prior knowledge of surgical robots; (3) mental representation of a surgical robot; (4) expectations of intraoperative robotic assistance; (5) emotions associated with its use prior to witnessing the robot demonstration; and (6) feedback after witnessing the robot demonstration. Questions 1–13 of the survey were filled out by participants before witnessing the robot demonstration, while questions 14–15 were filled out after, to capture participants’ perceptions before and after exposure to the robotic system.

**Figure 1:**
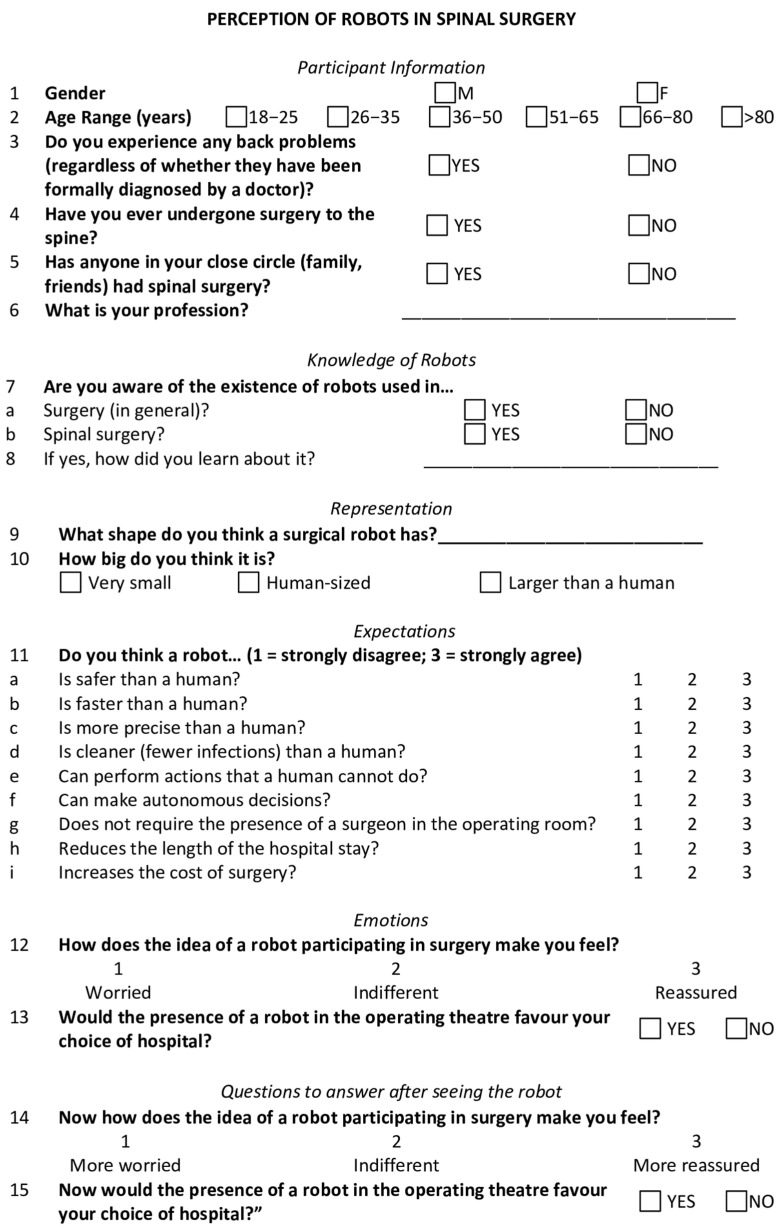
Study questionnaire, translated into English from the original Italian. The layout and font were intentionally preserved to accurately reflect the survey as presented to participants.

The initial draft was developed by the authors (L.F., A.M., and I.C.) based on expert input and iterative review. It was then evaluated by individuals from 5 different professional categories (1 neurosurgeon not involved in the study, 5 scrub nurses, 5 ward nurses, 5 medical secretaries, and 2 non-medical professionals) to assess its completeness, clarity, and ease of use. The final version of the questionnaire was revised based on the feedback received (Figure 1).

The data were collected anonymously and recorded in an Excel sheet for statistical analysis.

### 2.2. Statistical Analysis

Qualitative data were presented as absolute numbers with the corresponding percentages. The association between robot-specific random variables and subject-relevant variables was assessed using the chi-squared test. The impact of the robot’s public presentation on concerns about its use in surgery and the choice of undergoing surgery in a robot-equipped hospital was estimated using the Wilcoxon test and the McNemar test, respectively. To identify potential predictors of the choice to undergo surgery in a robot-equipped hospital, univariable logistic regression analyses were performed.

All statistical tests were two-sided, and a *p*-value of <0.05 was considered statistically significant. Statistical analysis was performed using Stata version 17.0 software (StataCorp LP, College Station, TX, USA).

## 3. Results

### 3.1. Demographics

A total of 109 questionnaires were collected. The responses are summarized in Table 1. Most participants were female (64.4%) and had no direct experience with spinal pathology: 79.8% reported no back problems, 96.3% had never undergone spine surgery, and 67.9% had no close acquaintances who had undergone spine surgery.

Participants’ occupations were categorized into the following fields: healthcare and social assistance, business and finance, education and academia, hospitality and tourism, construction and manual labor, government and public administration, science and research, technology and engineering, creative arts and media, and retired. The largest groups were retired individuals (25.8%) and healthcare workers (23.7%).

### 3.2. Knowledge, Expectations, and Concerns Regarding Robots in Spine Surgery

Most respondents were already aware of robotic technology in surgery: 87.2% knew about its general application in surgery, while 65.1% were specifically aware of its use in spine surgery. Participants’ replies concerning how they learnt about robot-assisted spine surgery were categorized into the following fields: media, work-related, and acquaintances. The primary source of information was the media (55.4%).

Regarding the mental representation of the robot, most participants described it as having one or more robotic arms (79.0%) and being approximately human-sized (45.7%). However, a quarter of participants imagined it as smaller than a human (26.7%), while another quarter perceived it as larger than a human (27.6%).

In terms of expectations, compared to a surgeon, the robot was perceived as faster (51.9%), more precise (70.4%), cleaner (54.6%), and capable of performing movements impossible for a human (50.9%). However, the majority believed that the robot could not make autonomous decisions (79.6%) or perform surgery without a surgeon present in the operating room (84.3%), and 67.9% did not consider the surgical robot to be safer than a human surgeon.

Trust in the robot’s capabilities was assessed before and after participants observed a live demonstration of the robotic spine system. Initially, 61.3% felt reassured by the presence of a robot during surgery, but this increased to 81.9% after the demonstration (*p* = 0.007). Similarly, before the demonstration, 50.5% stated they would prefer to be treated in a hospital using robotic systems for spinal surgery, rising to 64.5% afterward (*p* < 0.001).

Multivariable analysis found no significant association between sex, age, profession, and participants’ mental image of a surgical robot (questions 7–10), expectations of robotic surgery (questions 11a–11i), or trust in robotic surgery before and after the demonstration (questions 12–15). However, individuals with back-related problems were more likely to believe that robots could perform movements impossible for a human (*p* = 0.032) and that robot-assisted surgeries were less expensive (*p* = 0.036). No significant association was found between past spinal surgeries and expectations of the robot.

## 4. Discussion

### 4.1. Key Findings

To the best of our knowledge, this study is the first to investigate public perception of robot-assisted spine surgery. Our findings indicate that while most participants were aware of robotic surgery in general, fewer knew about its specific application in spine surgery, with media being the primary source of information.

Regarding the mental representation of spine surgery robots, most participants envisioned them as having one or more robotic arms and being human-sized, though perceptions varied.

Participants generally viewed the robot as more precise, cleaner, and capable of complex—but not autonomous—movements compared to a human surgeon; however, most did not consider it necessarily safer. Trust in robot-assisted spine surgery significantly increased after witnessing a live demonstration, as did the preference for surgery in a hospital equipped with robotic systems.

Multivariable analysis found no significant associations between demographic factors or profession and expectations, but individuals with back problems were more likely to attribute unique capabilities to the robot.

### 4.2. Comparison with Other Studies on Public Perception of Robotic Systems in Healthcare

Although data on the general public’s perception of robotic systems in spine surgery was previously lacking, a few studies—although limited in number—have explored public attitudes toward the introduction of robots in the medical field, revealing divergent views. Aymerich-Franch et al. [1] reported a generally positive attitude towards the implementation of robots in healthcare. Moreover, they found an association between female sex and religiousness with fear of robots. In contrast, our study did not observe a similar association between gender—or any other demographic factor—and expectations or concerns regarding robot-assisted spine surgery. Additionally, while our study did not assess the religiousness of our participants, it did examine their professions and found no association with expectations or emotions.

In contrast to Aymerich-Franch et al. [1], McDonnell et al. [2] found that the general public perceives robotic surgery as risky. The authors suggest that this perception may stem from the public’s non-expert understanding of the modality, potentially triggering fears of increased complications. Our cohort did not significantly reflect such fears, as only 6.6% of participants strongly disagreed with the statement that the spine surgery robot is safer than a human surgeon, while 61.3% remained neutral and one-third actually considered the robot to be safer. Participants also attributed other positive characteristics to the robot: half believed it to be faster, cleaner, and capable of more complex movements than a human surgeon, while more than two-thirds felt that it was more precise. Additionally, a significant portion (41.7%) believed its use was associated with a reduction in the length of hospital stays.

The fact that the vast majority of participants did not perceive the robot as autonomous, while still recognizing the advantages stated above, suggests that it is viewed as a tool able to refine the surgeon’s capabilities while remaining under human control. This is in line with findings of Aymerich-Franch et al. [1] that showed that public acceptance was higher for robots assisting a human rather than performing the role by itself.

In contrast, we found that individuals with back problems exhibited greater confidence in the robot, more often believing it could perform movements beyond human capability compared to participants without such issues—possibly due to dissatisfaction with available therapies for their chronic condition, leading to heightened expectations of emerging treatment modalities. They also assumed that robot-assisted surgery would be less expensive. This finding may again be explained by their elevated expectations of this novel technology, alongside their presumed increased awareness of healthcare costs, given that Swiss patients typically receive their medical bills directly and contribute personally up to a given threshold.

### 4.3. Implications

Although participants generally attributed positive baseline characteristics to the spine surgery robot, we observed that trust in the robot further increased after they viewed a live demonstration and interacted with it (61.3% before vs. 81.9% after, *p* = 0.007). Such interactions likely facilitate a better understanding of the robot’s technical capabilities and advantages, while also defusing fearful misrepresentations. Furthermore, our findings suggest that opportunities to interact with the robot also positively influence participants’ preference for hospitals offering robot-assisted surgery. This observation can be perceived as a marketing incentive for robot-naïve hospitals to acquire this technology, and for hospitals already equipped with a spine surgery robot to actively showcase it to the public. However, it also raises concerns about the impact of selective promotion in relation to other enabling technologies, such as non-robotic spinal navigation, which has demonstrated its clinical utility [5] and is reported to have an accuracy similar to that of robotic assistance [11]. Additionally, the overridingly critical role of surgical skill and decision-making may be overshadowed.

### 4.4. Study Strengths and Limitations

The questionnaire was developed through an iterative review process involving individuals with and without a healthcare background to ensure comprehensiveness and user-friendliness. To minimize hesitation in questions requiring a quantitative assessment of feelings, a three-item response scale was used instead of the five-item Likert scale, as non-experts may find it difficult to express varying degrees of opinions on such a specific topic.

Certain limitations should be considered when interpreting this study’s findings. Firstly, while the decision to conduct this survey during an open day for the general public aimed to recruit a diverse and representative sample of the general population, the participant pool was relatively limited (N = 109) and may have been subject to a selection bias as attendees were likely predisposed to an interest in our Neurocenter and may not reflect broader public perspectives. Although the event was open to the general public, a significant portion of participants (23.7%) worked in healthcare, and the largest group (25.8%) was retired. This suggests that younger individuals may have been less inclined to attend, potentially limiting the generalizability of our results across broader age demographics. Additionally, participants’ education level may also affect generalizability. While we had initially intended to investigate the influence of education level, we decided to collect profession data as a surrogate to avoid making participants feel uncomfortable with potentially intrusive questions. We found that profession was not significantly associated with participants’ representations of the surgical robot, or with their trust in the robotic system before and after the demonstration. However, one-quarter of participants reported being “retired” without specifying their previous profession, which limits the analysis.

Furthermore, age was surveyed as a categorical rather than a continuous variable to classify participants by generation and reduce potential discomfort in disclosing their exact age.

The results of this survey provide insights into the perception of robot assistance in spine surgery. However, larger studies are needed to confirm the present findings. Notably, our study suggests that the availability of a spinal robot may positively influence patients’ choice of hospital for undergoing surgery. However, this may also lead to diminished consideration of alternative methods, such as computer-assisted navigation, despite their demonstrated efficacy in pedicle screw placement.

## 5. Conclusions

Spine surgery is a rapidly evolving field driven by technological advancements, and the adoption of surgical robots is a key consideration for both surgeons and hospital administrators, who must weigh the benefits of this technology against its high costs, in the presence of already well-established and effective alternatives such as computer-assisted navigation. Patient perception also plays a crucial role in this process; yet, to our knowledge, this is the first such study in the context of robot-assisted spine surgery.

This study suggests that the general population holds a positive attitude toward this technology, viewing robots as faster, more precise, cleaner, and capable of performing movements beyond human ability. We found that people felt reassured by the presence of a robot in the operating theater, and this confidence was further strengthened after witnessing the robot in action. Moreover, after a live demonstration of the spinal robot, participants expressed a preference for treatment in a hospital that uses this technology over one that has not implemented it. This finding has economic implications for hospitals and also highlights the importance of exposing the general public to live demonstrations of new surgical technologies for reassurance in the face of novelty, while also prompting necessary reflection on the influence of patient perception bias on decision-making and the need to maintain a critical perspective on the benefits of new surgical techniques to mitigate that bias.

## Figures and Tables

**Table 1:** Distribution of participants’ responses to the questionnaire.

	*n*	%
Sex		
M	36	35.6
F	65	64.4
Age		
18–25	11	10.1
26–35	15	13.7
36–50	38	34.9
51–65	29	26.6
66–80	15	13.8
80+	1	0.9
Back problems		
Yes	22	20.2
No	87	79.8
Previous spine surgery		
Yes	4	3.7
No	104	96.3
Spine surgery in acquaintances		
Yes	34	32.1
No	72	67.9
Job		
Healthcare and social assistance	22	23.7
Business and finance	8	8.6
Education and academia	10	10.7
Hospitality and tourism	2	2.1
Construction and manual labor	13	14.0
Government and public administration	9	9.7
Science and research	1	1.1
Technology and engineering	3	3.2
Creative arts and media	1	1.1
Retired	24	25.8
Knowledge of robot in surgery (general)		
Yes	95	87.2
No	14	12.8
Knowledge of robot in spine surgery		
Yes	69	65.1
No	14	34.9
Source of information		
Media	41	55.4
Work-related	21	28.4
Acquaintances	12	16.2
Shape		
Robotic arm(s)	49	79.0
Computer box	1	1.6
Complex machinery	9	14.5
Other	3	4.9
Size		
Smaller than a human	28	26.7
Human-sized	48	45.7
Bigger than a human	29	27.6
Expectations		
Safer		
1	7	6.6
2	65	61.3
3	34	32.1
Faster		
1	9	8.3
2	43	39.8
3	56	51.9
More precise		
1	5	4.6
2	27	25.0
3	76	70.4
Cleaner		
1	10	9.3
2	39	36.1
3	59	54.6
Gestures impossible for a human		
1	18	16.7
2	35	32.4
3	55	50.9
Autonomous decisions		
1	86	79.6
2	15	13.9
3	7	6.5
Independent		
1	91	84.3
2	11	10.2
3	6	5.6
Reduction in length of hospital stays		
1	20	18.5
2	43	39.8
3	45	41.7
Increased costs of surgery		
1	24	22.2
2	52	48.2
3	32	29.6
Before seeing robot		
Reassured by the presence of robot		
1	9	8.5
2	32	30.2
3	65	61.3
Choice of the hospital with robot		
Yes	54	50.5
No	53	49.5
After seeing robot		
Reassured by the presence of robot		
1	2	1.9
2	17	16.2
3	86	81.9
Choice of the hospital with robot		
Yes	69	64.5
No	38	35.5

## Data Availability

The raw data supporting the conclusions of this article will be made available by the authors on request.
